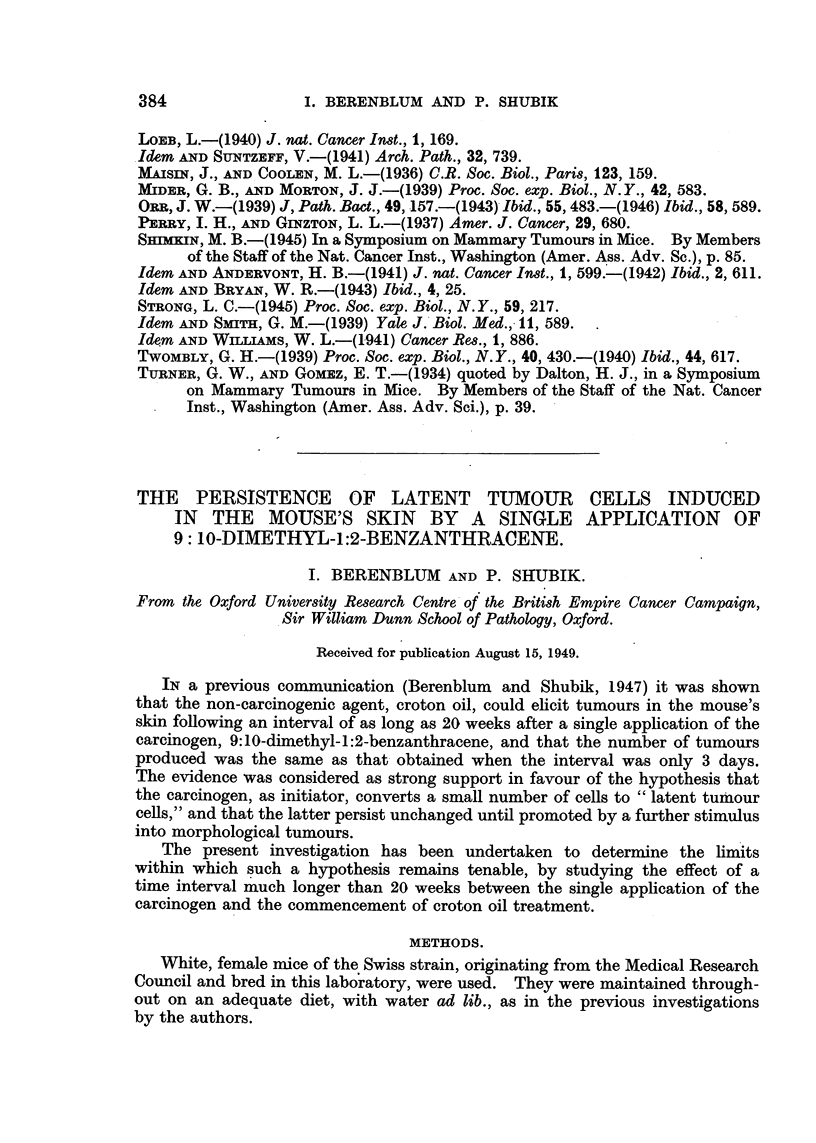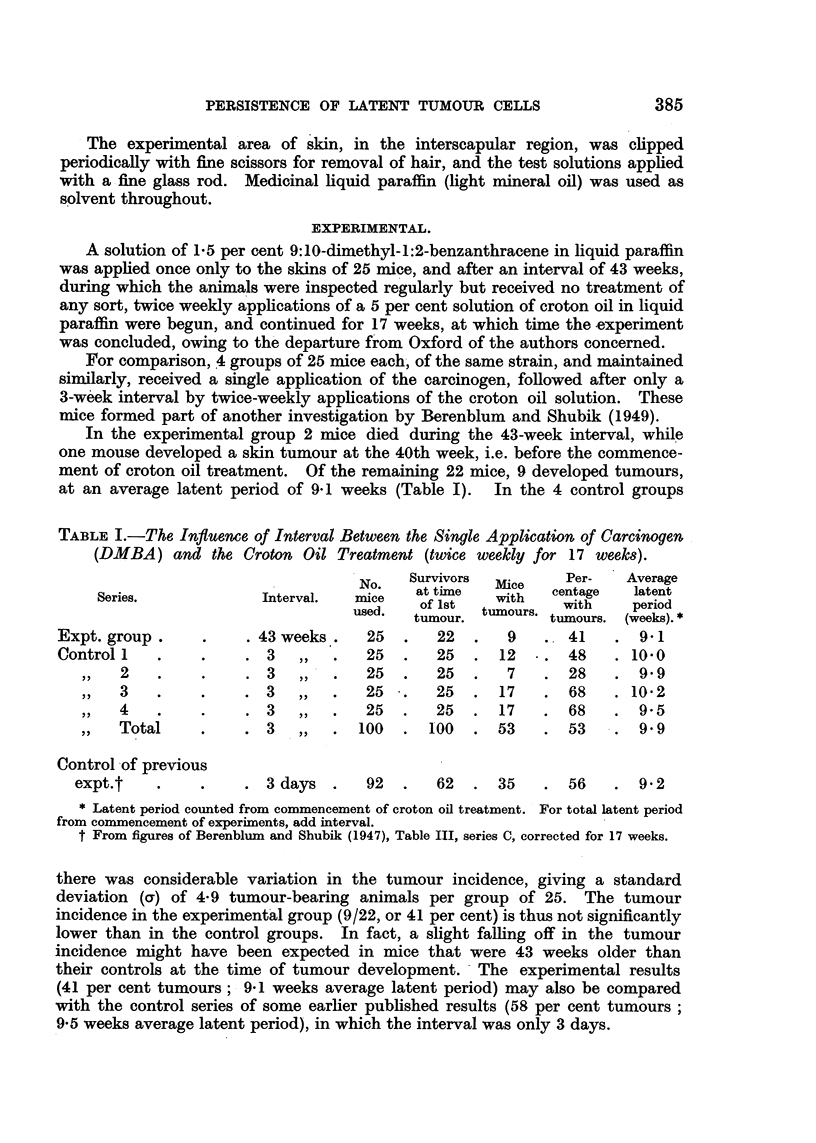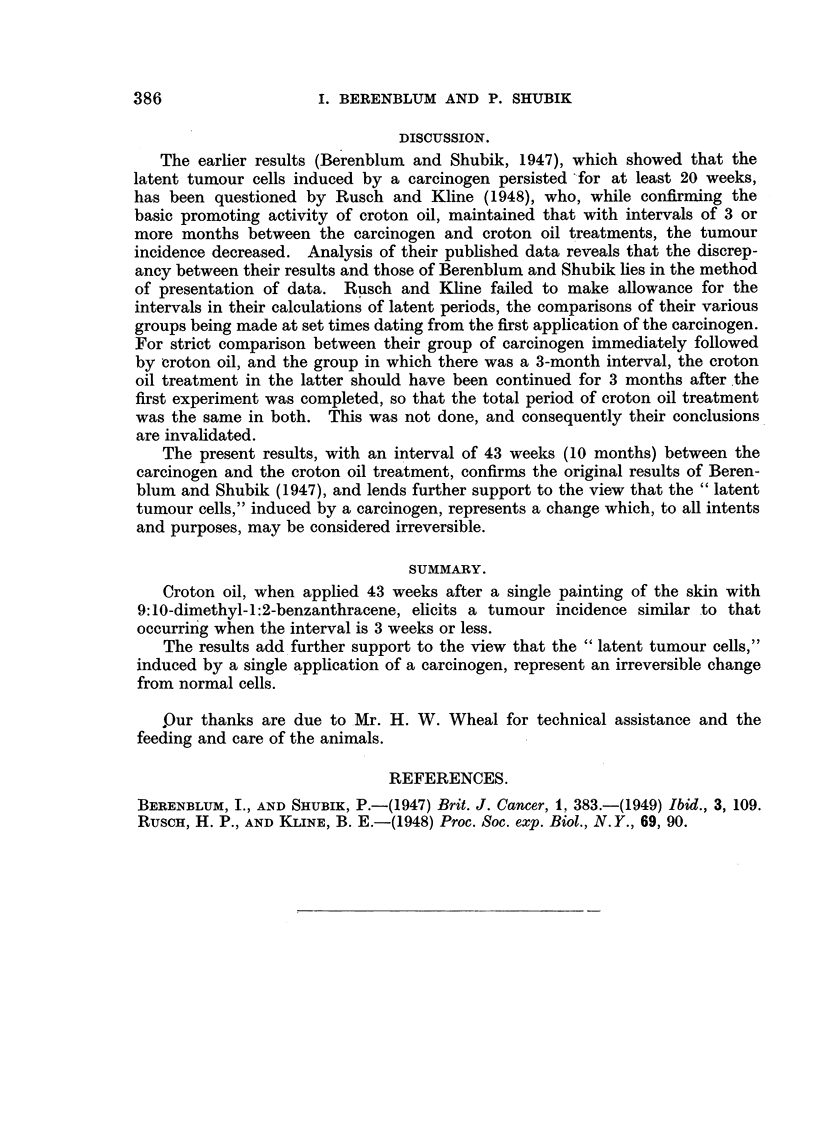# The Persistence of Latent Tumour Cells Induced in the Mouse's Skin by a Single Application of 9:10-Dimethyl-1:2-Benzanthracene

**DOI:** 10.1038/bjc.1949.42

**Published:** 1949-09

**Authors:** I. Berenblum, P. Shubik


### THE PERSISTENCE OF LATENT TUMOUR CELLS INDUCED IN THE MOUSE'S SKIN BY A SINGLE APPLICATION OF 9:10-DIMETHYL-1:2-BENZANTHRACENE.

I. BERENBLUM AND P. SHUBIK.

*From the Oxford University Research Centre of the British Empire Cancer Campaign, Sir William Dunn School of Pathology, Oxford.*

Received for publication August 15, 1949.

IN a previous communication (Berenblum and Shubik, 1947) it was shown that the non-carcinogenic agent, croton oil, could elicit tumours in the mouse's skin following an interval of as long as 20 weeks after a single application of the carcinogen, 9:10-dimethyl-1:2-benzanthracene, and that the number of tumours produced was the same as that obtained when the interval was only 3 days. The evidence was considered as strong support in favour of the hypothesis that the carcinogen, as initiator, converts a small number of cells to " latent tumour cells," and that the latter persist unchanged until promoted by a further stimulus into morphological tumours.

The present investigation has been undertaken to determine the limits within which such a hypothesis remains tenable, by studying the effect of a time interval much longer than 20 weeks between the single application of the carcinogen and the commencement of croton oil treatment.

### METHODS.

White, female mice of the Swiss strain, originating from the Medical Research Council and bred in this laboratory, were used. They were maintained throughout on an adequate diet, with water *ad lib.*, as in the previous investigations by the authors.

The experimental area of skin, in the interscapular region, was clipped periodically with fine scissors for removal of hair, and the test solutions applied with a fine glass rod. Medicinal liquid paraffin (light mineral oil) was used as solvent throughout.

### EXPERIMENTAL.

A solution of 1·5 per cent 9:10-dimethyl-1:2-benzanthracene in liquid paraffin was applied once only to the skins of 25 mice, and after an interval of 43 weeks, during which the animals were inspected regularly but received no treatment of any sort, twice weekly applications of a 5 per cent solution of croton oil in liquid paraffin were begun, and continued for 17 weeks, at which time the experiment was concluded, owing to the departure from Oxford of the authors concerned.

For comparison, 4 groups of 25 mice each, of the same strain, and maintained similarly, received a single application of the carcinogen, followed after only a 3-week interval by twice-weekly applications of the croton oil solution. These mice formed part of another investigation by Berenblum and Shubik (1949).

In the experimental group 2 mice died during the 43-week interval, while one mouse developed a skin tumour at the 40th week, i.e. before the commencement of croton oil treatment. Of the remaining 22 mice, 9 developed tumours, at an average latent period of 9·1 weeks (Table I). In the 4 control groups

TABLE I.—*The Influence of Interval Between the Single Application of Carcinogen (DMBA) and the Croton Oil Treatment (twice weekly for* 17 *weeks).*

| Series. | Interval. | No. mice used. | Survivors at time of 1st tumour. | Mice with tumours. | Percentage with tumours. | Average latent period (weeks).* |
|---|---|---|---|---|---|---|
| Expt. group | 43 weeks | 25 | 22 | 9 | 41 | 9·1 |
| Control 1 | 3 ,, | 25 | 25 | 12 | 48 | 10·0 |
| ,, 2 | 3 ,, | 25 | 25 | 7 | 28 | 9·9 |
| ,, 3 | 3 ,, | 25 | 25 | 17 | 68 | 10·2 |
| ,, 4 | 3 ,, | 25 | 25 | 17 | 68 | 9·5 |
| ,, Total | 3 ,, | 100 | 100 | 53 | 53 | 9·9 |
| Control of previous expt.† | 3 days | 92 | 62 | 35 | 56 | 9·2 |

* Latent period counted from commencement of croton oil treatment. For total latent period from commencement of experiments, add interval.

† From figures of Berenblum and Shubik (1947), Table III, series C, corrected for 17 weeks.

there was considerable variation in the tumour incidence, giving a standard deviation ($\sigma$) of 4·9 tumour-bearing animals per group of 25. The tumour incidence in the experimental group (9/22, or 41 per cent) is thus not significantly lower than in the control groups. In fact, a slight falling off in the tumour incidence might have been expected in mice that were 43 weeks older than their controls at the time of tumour development. The experimental results (41 per cent tumours ; 9·1 weeks average latent period) may also be compared with the control series of some earlier published results (58 per cent tumours ; 9·5 weeks average latent period), in which the interval was only 3 days.

### DISCUSSION.

The earlier results (Berenblum and Shubik, 1947), which showed that the latent tumour cells induced by a carcinogen persisted for at least 20 weeks, has been questioned by Rusch and Kline (1948), who, while confirming the basic promoting activity of croton oil, maintained that with intervals of 3 or more months between the carcinogen and croton oil treatments, the tumour incidence decreased. Analysis of their published data reveals that the discrepancy between their results and those of Berenblum and Shubik lies in the method of presentation of data. Rusch and Kline failed to make allowance for the intervals in their calculations of latent periods, the comparisons of their various groups being made at set times dating from the first application of the carcinogen. For strict comparison between their group of carcinogen immediately followed by croton oil, and the group in which there was a 3-month interval, the croton oil treatment in the latter should have been continued for 3 months after the first experiment was completed, so that the total period of croton oil treatment was the same in both. This was not done, and consequently their conclusions are invalidated.

The present results, with an interval of 43 weeks (10 months) between the carcinogen and the croton oil treatment, confirms the original results of Berenblum and Shubik (1947), and lends further support to the view that the " latent tumour cells," induced by a carcinogen, represents a change which, to all intents and purposes, may be considered irreversible.

### SUMMARY.

Croton oil, when applied 43 weeks after a single painting of the skin with 9:10-dimethyl-1:2-benzanthracene, elicits a tumour incidence similar to that occurring when the interval is 3 weeks or less.

The results add further support to the view that the " latent tumour cells," induced by a single application of a carcinogen, represent an irreversible change from normal cells.

Our thanks are due to Mr. H. W. Wheal for technical assistance and the feeding and care of the animals.

### REFERENCES.

BERENBLUM, I., AND SHUBIK, P.—(1947) *Brit. J. Cancer*, **1**, 383.—(1949) *Ibid.*, **3**, 109.
RUSCH, H. P., AND KLINE, B. E.—(1948) *Proc. Soc. exp. Biol., N.Y.*, **69**, 90.